# Microstructure and Mechanical Properties of Ultrafine-Grained Copper by Accumulative Roll Bonding and Subsequent Annealing

**DOI:** 10.3390/ma13225171

**Published:** 2020-11-16

**Authors:** Xueran Liu, Limin Zhuang, Yonghao Zhao

**Affiliations:** 1 Nano and Heterogeneous Materials Center, School of Materials Science and Engineering, Nanjing University of Science and Engineering, Nanjing 210094, China; xrliu@ycit.cn (X.L.); zhuang.limin@cxtc.com (L.Z.); 2 School of Materials Science and Engineering, Yancheng Institute of Technology, Yancheng 224051, China

**Keywords:** accumulative roll bonding, ultrafine-grained Cu, annealing, microstructure, mechanical properties

## Abstract

Recently, the accumulative roll bonding (ARB) technique has made significant progress in the production of various ultrafine-grained (UFG) metals and alloys. In this work, a UFG copper sheet was produced by ARB and subsequent annealing at 300 °C for 60 min to optimize strength and ductility. It was found that homogeneous lamellar UFG materials with a thickness of 200–300 nm were formed after six ARB passes. The microhardness and tensile strength of as-ARBed Cu increased, while the ductility and strain hardening decreased with the cumulative deformation strain. The as-ARBed specimens fractured in a macroscopically brittle and microscopically ductile way. After annealing, discontinuous recrystallization occurred in the neighboring interface with high strain energy, which was prior to that in the matrix. The recrystallization rate was enhanced by increasing the cumulative strain. UFG Cu ARBed for six passes after annealing manifested a completely recrystallized microstructure with grain sizes approximately ranging from 5 to 10 μm. Annealing treatment reduced the microhardness and tensile strength but improved the ductility and strain hardening of UFG Cu. As-annealed UFG-Cu fractured in a ductile mode with dominant dimples and shear zones. Our work advances the industrial-scale production of UFG Cu by exploring a simple and low-cost fabrication technique.

## 1. Introduction

Worsening energy crisis and greenhouse effect have prompted the study of high-strength metals and alloys for critical applications in the aerospace, transportation, and biomedical industries, because when properly designed in engineering systems, these materials can lead to reductions in greenhouse gases, such as CO_2_. Results over the past few decades suggest that it is possible to obtain significant (5–10 times) increases in the strength of bulk nanostructured (10–100 nm grain size, NS) and/or ultrafine-grained (100–1000 nm grain size, UFG) metals and alloys, which have prompted numerous studies [1,2]. The high strength of bulk NS and UFG materials results from the high density of lattice defects, such as grain boundaries (GBs), triple boundaries, and dislocations. Grain refinement via severe plastic deformation (SPD) techniques [3] is a promising route for preparing different NS and UFG materials, especially for pure metals, for a variety of applications, such as in chemical, metallurgical, and biological engineering and the nuclear industry. Compared with conventional coarse-grained (CG) materials, except those with high strength, NS and UFG metallic materials exhibit higher hardness, superior room-temperature corrosion resistance, and low-temperature superplasticity [4,5,6,7]. Up to now, many different SPD techniques are being developed, including equal-channel angular pressing (ECAP) [8], high-pressure torsion (HPT) [9], surface mechanical attrition treatment (SMAT) [10], rotary swaging (RS) [11], multidirectional forging (MDF) [12], high-ratio differential speed rolling [13], large-strain extrusion machining [14], accumulative roll bonding (ARB) [7], quasi-static deformation (QSD), and cryorolling [2,3]. Most of the SPD techniques above are effective in grain refinement, and some of them can even refine the grain size down to nanometer scale [9,10,11]. However, the sample size processed by SPD is normally of the centimeter or even millimeter scale and difficult to scale further up, thereby limiting their applications in the industry. For example, the sample processed by HPT is usually 1 mm thick [9], and the sample surface treated by SMAT is 10 micrometers thick [10]. Moreover, the current SPD techniques have the disadvantages of complexity, high cost, and low efficiency. For instance, both HPT and ECAP need high pressure [8,9], and RS and MDF require dynamic/high-speed deformation [11,12]. Among the different SPD techniques, ARB is the one that is a ready-made deformation technique in the industry; therefore, the preparation of UFG materials by ARB has a simple operation, low cost, and easy commercialization [7]. Moreover, the size of the sample processed by ARB is not limited and can easily meet the needs of industry applications. In theory, ARB can be rolled repeatedly without restrictions; therefore, the grain size can be easily refined down to the UFG region.

Recently, the accumulative roll bonding (ARB) technique has made significant progress in the production of various UFG metallic materials, such as pure metals [15,16,17,18], alloys [19,20,21], and particles reinforced metal matrix composites [22,23], with desired microstructure and superior mechanical properties. Theoretically, ARB can achieve grain refinement through endless roll bonding because of extremely high strains. For instance, Saito et al. [15] employed ARB to produce UFG AA 1100 with a grain size of less than 1 μm. Moreover, the average grain size of ARB-fabricated AA 1050 reaches as little as 380 nm after eight passes [24]. Furthermore, ARB-processed OFHC-Cu has exhibited an average grain size of 200–300 nm [17]. Similarly, ARB-processed IF steel has been shown to have an average grain size of 200 nm after five ARB passes [18].

Subsequently, the strengths of ARB-processed UFG materials remarkably increase due to grain refinement and strain hardening. For instance, ARB-processed UFG materials have exhibited two/four times higher strength than their CG counterparts [25,26]. However, UFG materials exhibit high strength but poor ductility at room temperature, which limits their potential application in the industry. The research shows that low work hardening is the main factor limiting the plasticity of UFG metals [27]. Therefore, gradient materials and heterogeneous structures are used to improve the plasticity of materials [27,28,29,30,31]. The annealing of ARB-processed UFG metals is also carried out to enhance their ductility. For instance, Kwan et al. [28,29] demonstrated that an optimal annealing process can enhance the ductility of ARB-processed Al 1100 without sacrificing much of its strength. Morovvai et al. [30] reported that ARB-processed Al 1200 alloy exhibits a brittle fracture, whereas annealed ARB-processed Al 1200 alloy shows a ductile fracture. Similarly, Karimi et al. [31] showed that ARB-annealed Ti/SiC composites exhibit higher uniform elongation than ARB-processed Ti/SiC due to higher work hardening. Moreover, ultrafine grains have shown partial or total transformation into recrystallized grains after annealing, leading to a bimodal grain size distribution with ultrafine and micron-sized grains. The bimodal microstructure renders an optimal combination of strength and ductility [27]. Thus, the above results indicate that the mechanical properties of ARB-processed UFG metals can be finely tuned by optimizing the processing and annealing parameters.

Herein, UFG Cu was prepared through ARB processing for different passes, and then the ARB-processed specimens were annealed at 300 °C for 60 min in order to obtain a different progress of recovery and recrystallization. It was observed that the bimodal microstructure, with deformed and recrystallized grains, rendered an optimal combination of strength and ductility. The influence of the ARB process parameters and annealing on the microstructural evolution and mechanical properties of UFG Cu was systematically investigated.

## 2. Experimental Procedures

### 2.1. Fabrication of UFG Cu

Commercially available oxide-free high-conductivity (OFHC) high-purity (99.98%) Cu was used in this work. Before ARB processing, an as-received Cu sheet with a thickness of 1 mm was sectioned into 150 × 25 mm strips and then annealed at 450 °C for 120 min in an argon atmosphere to obtain a completely recrystallized microstructure with an average grain size of about 50 μm. For ARB processing, the surface of annealed Cu strips was first degreased with acetone and then polished with a rotating wire brush. Then, two pieces of Cu strips were stacked together and riveted with steel nails at four corners. Finally, the stacked and riveted Cu strips were rolled by a rolling mill with a roll diameter of 120 mm and a rolling speed of 0.34 m/s without lubrication. After the previous rolling pass, the as-rolled specimens were sectioned into two halves and stacked and riveted together again for subsequent rolling. In each rolling pass, the thickness of the stacked Cu strips was reduced by 50%, corresponding to a von Mises equivalent strain ε of 0.8. The final rolling pass was up to six passes with ε = 4.8 (Table 1). 

To optimize their mechanical properties, ARB-processed specimens were annealed at 300 °C for 60 min under Ar atmosphere to obtain an optimal combination of strength and ductility.

### 2.2. Microstructural Characterization

**Figure 1:**
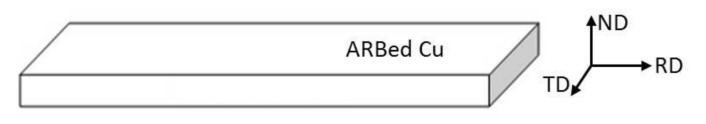
Schematic representation of the ARBed Cu plates with RD, ND, and TD coordinate systems. RD—rolling direction, ND—normal direction, TD—tran sverse direction.

Microstructural characterization was carried out by means of electron backscatter diffraction (EBSD) and transmission electron microscopy (TEM) techniques. EBSD and TEM observations were performed on the rolling direction–normal direction (RD–ND) planes of the as-ARBed and as-annealed specimens, as schematically shown in Figure 1. Specifically, EBSD mapping of ARBed specimens was conducted using a high-resolution field emission Carl Zeiss-Auriga-45–66 scanning electron microscope (SEM) in a FEI Quanta 250 F device equipped with a fully automatic Oxford Instruments Aztec 2.0 EBSD system (Channel 5 software) operating at 20 kV. The EBSD specimens were mechanically polished with SiC paper and subsequently electropolished in an electrolyte containing 85 vol. % phosphoric acid and 15 vol. % deionized water using a voltage of 35 V and a polishing time of 50 s in a Buehler ElectroMet 4 polisher (Buehler Ltd., Lakbluff, IL, USA). In EBSD maps, the black thick lines denote the high-angle grain boundaries (HAGBs) with a misorientation angle more than 15°, and the gray thin lines represent the low-angle grain boundaries (LAGBs) with a misorientation angle between 2° and 15°.

TEM observations were conducted in a FEI-Tecnai G^2^ 20 S-TWIN microscope (FEI, Hillsboro, OR, USA) operated at 200 kV. The TEM specimens were prepared by grinding the deformed specimens down to 60 μm thickness and then punched into discs with a diameter of 3 mm. The discs were then dimpled to a thickness of about 10 μm, and finally ion-milled to a thickness of electron transparency using a Gatan Precision Ion Milling System with an Ar+ accelerating voltage of 4 kV at a temperature below 35 °C. The microstructures of the annealed specimens were examined with a Zeiss Auriga CrossBeam system (FIB/SEM, Zeiss, Oberkochen, Germany) operating at 5 kV.

### 2.3. Mechanical Properties

Vickers microhardness was measured by using an HMV-G 21DT apparatus (Shimadzu, Kyoto, Japan) under a load of 0.49 N for 15 s. In the RD–ND plane, 10 values of microhardness are measured with equally spaced 10 points. Then, the maximum and minimum values are discarded, and the remaining 8 values are averaged out to report the microhardness of the sample.

Room-temperature tensile testing was carried out by using a Walter + bai LFM 20 kN tensile machine (Walter + bai ag, Schaffhausen, Switzerland) at an initial strain rate of 5.6 × 10^−4^ s^−1^. The tensile direction was parallel to the rolling direction. The gauge length, width, and thickness of the tensile specimens were 15, 2, and 1 mm, respectively. In addition, the fractured surface of the tensile specimens was observed using FEI Quanta 250F SEM (FEI, Hillsboro, OR, USA).

## 3. Results

### 3.1. Microstructure of ARB-Processed Cu

#### 3.1.1. Grain Size and GBs

**Figure 2:**
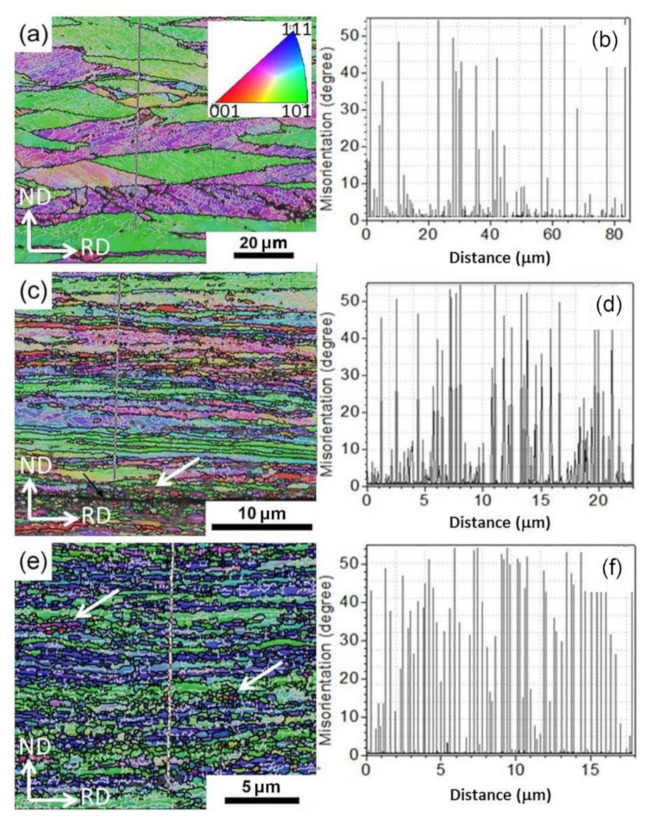
EBSD crystal orientation inverse pole figure (IPF) maps (**a**,**c**,**e**) and corresponding misorientation distributions (**b**,**d**,**f**) along the lines from top to bottom of ARBed Cu with (**a**,**b**) 1 pass, (**c**,**d**) 3 passes, and (**e**,**f**) 6 passes. The inset is color coded, in which red, green, and blue indicate grains having <001>, <101>, and <111> directions perpendicular to the plane, respectively. The gray lines represent the LAGBs with a misorientation angle θ of 2° ≤ θ < 15°, and the black lines represent HAGBs with a misorientation of θ *>* 15°. The bin size of the misorientation distributions Δθ is 2°.

The microstructural evolutions, including grain size and morphology, GB misorientation angle distribution, and texture of Cu during ARB processing, were achieved in the EBSD analysis. The typical EBSD crystal orientation inverse pole figure (IPF) maps of ARBed Cu with one pass, three passes, and six passes and the corresponding misorientation distributions along the lines from top to bottom are shown in Figure 2. The gray lines represent the LAGBs with a misorientation angle θ of 2° ≤ θ < 15°, and the black lines represent high-angle grain boundaries (HAGBs) with a misorientation of θ *>* 15°. The bin size of the misorientation distributions Δθ is 2°. After one pass of ARB, the initial CGs were refined in the ND direction and elongated significantly along the RD direction, as shown in Figure 2a. Moreover, the elongated CG grains are surrounded by HAGBs (black lines) and full of LAGBs (gray lines) at their interiors, as shown in Figure 2a. Figure 2b shows the quantitative misorientation variations from top to bottom along the line in Figure 2a, and further verified the above analysis (i.e., a large number of LAGBs were introduced between the initial HAGBs). As the number of ARB passes increased to three, the thickness of elongated grains in the ND direction was reduced significantly down to micrometer or even submicrometer scales (Figure 2c). Moreover, the numbers of both HAGBs and LAGBs increased, as shown in Figure 2d. There is an interface in Figure 2c without reaching metallurgical bonding yet, as pointed out by the black arrow, and the grain size near the interface is much smaller than that in the matrix.

After six ARB passes, the length of the elongated UFGs along the RD direction was shorter than that after three ARB passes because the elongated grains were broken by numerous equiaxed UFG grains, as pointed out by the white arrows, which might be formed via dynamic recrystallization (DRX) during the ARB process. As shown in Figure 2c,e, the grains pointed by the white arrows are equiaxed and have high-angle orientation with the Cu matrix (evident color difference between DRX grains and Cu matrix), which are characteristics of DRX grains. This suggests that restoration occurs to some degree during the ARB process, which can be attributed to accumulated strain and adiabatic heating due to large plastic deformation [32,33]. Higher accumulated strain after six passes corresponds to larger driving force for recovery and short-range GB migration. Figure 2f also verifies that numerous HAGBs with submicrometer interval appeared, which had a larger fraction than LAGBs. Moreover, the spacing of the HAGBs was quite homogeneous throughout the thickness of ARB-processed Cu for six passes. The average boundary spacing along the ND direction was in the range of 200 to 300 nm. However, some grains still possessed certain amounts of LAGBs.

**Figure 3:**
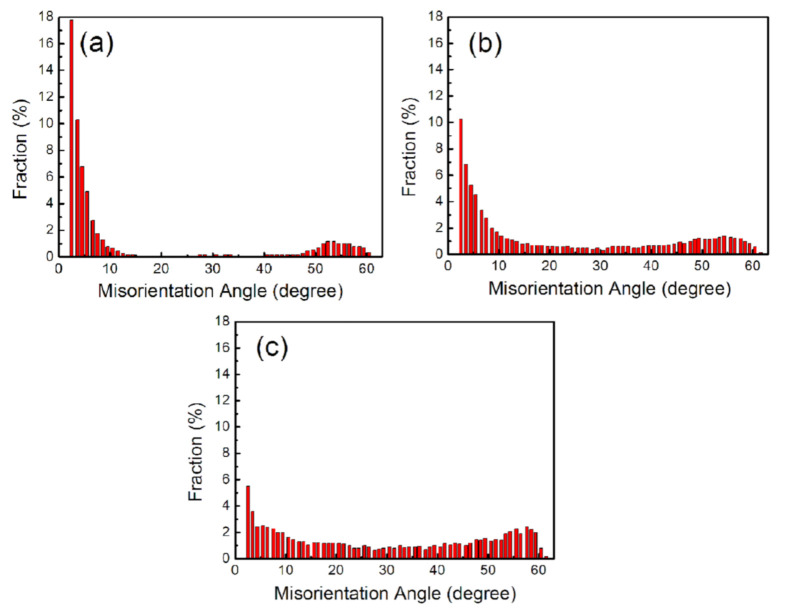
Distributions of boundary misorientation angles of Cu ARB-processed for (**a**) 1 pass, (**b**) 3 passes, and (**c**) 6 passes.

Figure 3 shows the quantitative distributions of the GB misorientation angles of as-ARBed Cu with one, three, and six passes. There are two peaks in the boundary misorientation distribution corresponding to large misorientation angle (45°–60°) and small misorientation angle (2°–15°). The bimodal distribution was observed in heavily deformed metals with medium stacking fault energy [23,34,35]. By increasing the number of ARB passes from one to six, the fraction of the LAGBs decreases and the fraction of the HAGBs increases. When the ARB pass number is six, more than 70% of the GBs can be categorized as HAGBs (Figure 3c).

#### 3.1.2. Texture Evolutions

Texture is usually expressed in polar and inverse polar (projection space) figures and Eulerian space and Rodrigues vector space. The miller index, Euler angle, and Rodrigues vector corresponding to the above space can be read from the orientation data and used to analyze the micro texture of the samples. Table 2 lists {100}, {110}, and {111} pole figures of ARB-processed Cu for different passes. In all pole figures, the *x*-axis of the sample coordinate system in the polar diagram corresponds to the RD direction, and the *y*-axis of the sample coordinate system corresponds to the ND direction. A weak texture occurs in ARB-processed Cu for one pass, which is a cube component reported by Shaarbaf et al. [32]. When the number of ARB passes increases, the intensity of the texture is enhanced. After six passes, the texture strength is basically stable. Moreover, the patterns formed by the poles projected on the polar map by stereographic projection have good correspondence. Therefore, although the texture strength inside the sample changes correspondingly after different passes of rolling, the texture type remains basically unchanged.

**Figure 4:**
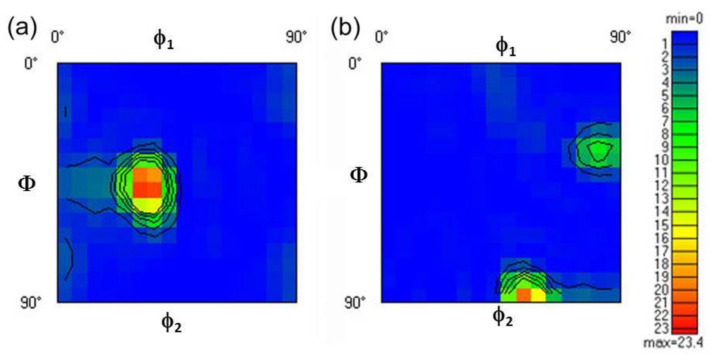
Orientation distribution function (ODF) of ARB-processed Cu for 6 passes when φ_2_ is 0° (**a**) and 45° (**b**).

In view of the sample symmetry and crystal symmetry of the sample, the Eulerian space expansion diagram (φ_2_ = 0° and φ_2_ = 45°) is used to identify the main texture components in the sample. As shown in Figure 4, the texture corresponding to the Euler angles (30°, 45°, 0°) and (52°, 86°, 45°) were the main texture components of ARB-processed Cu for six passes. The Euler angle representation of the above textures is equivalent to the miller index forms, which are (011) [2,3,4,5,6,7,8,9,10,11] and (110) [1,2,3,4,5,6,7,8,9,10,11,12], respectively. The main texture components of these two groups belong to {110} < 112 > rolling texture (i.e., {110}//RD–ND plane and <112>//RD direction). The above results show that the rolling texture of the Cu plate is mainly a Bs texture.

#### 3.1.3. Grains and Dislocations by TEM

**Figure 5:**
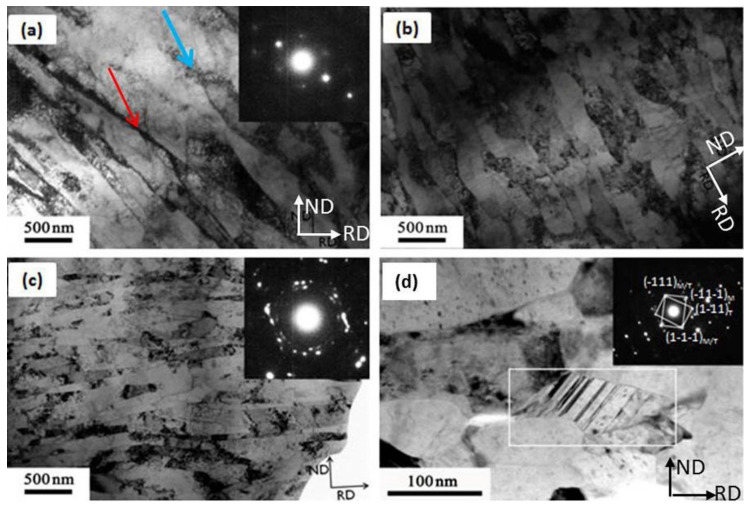
Bright-field TEM images of pure Cu after different ARB passes, (**a**) 1P, (**b**) 3P, (**c**), and (**d**) 6P; the selected area electron diffraction (SAED) patterns in (**a**,**c**) were obtained from a circle region of 750 nm in diameter, and the SAED pattern in (**d**) was obtained from a circle region of 200 nm in diameter.

Figure 5a–d shows the TEM micrographs of as-ARBed Cu with one to six passes at the RD–ND plane. After one pass rolling, multiple slip systems start simultaneously, and a large number of dislocations accumulate, forming dislocation entanglement structures with high dislocation density (pointed by the blue arrow in Figure 5a) and elongated substructures involving dislocation cells and subgrains with LAGBs, as pointed out by the red arrow in Figure 5a. The cell structures have the majority of dislocations tangling in the cell walls and a fairly low density of dislocations in cell interiors as a result of extensive dynamic recovery during the ARB process. Because the deformation strain (i.e., the von Mises equivalent strain of one pass) is small (0.8), the elongated cellular structure formed by individual dislocation tangles is not parallel with but forming a certain angle (40° ± 5°) to the rolling direction, as shown in Figure 5a. The average cell thickness ranges from 300 to 800 nm. The selected area electron diffraction (SAED) pattern shows that the cell boundaries are mainly small angle GBs.

Once the von Mises equivalent strain is increased to 2.4 (i.e., ARB process for three passes), a large number of geometrically necessary dislocations are introduced into the deformed microstructure to maintain the strain compatibility. The lamellar UFGs are formed along the rolling direction, and the fraction of the HAGBs is improved, as shown in Figure 5b. After six passes of cumulative rolling, the UFG lamellar grains have sharp GBs and low dislocation density in the grains due to dislocation recovery and DRX, as shown in Figure 5c. The lamellar grain thickness is about 200 nm. The SAED pattern shows a tendency of a continuous diffraction pattern, suggesting there exist both HAGBs and LAGBs. A small number of deformation twins were found, as shown in Figure 5d, which indicates that the grain refinement is mainly dominated by dislocation slip segmentation in the process of accumulative rolling deformation. When the grain is refined down to UFG regime, deformation twinning occurs.

**Figure 6:**
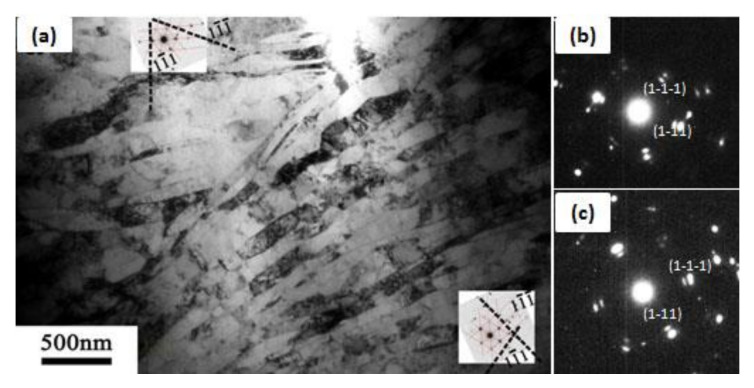
Bright-field TEM image (**a**) of pure Cu after 6 ARB passes: (**b**) and (**c**) are the corresponding SAED patterns of the upper-left corner and lower-right corner in (**a**), respectively. The SAED patterns were obtained from a circle region with 750 nm in diameter.

In addition, we found that not all UFG lamellae are parallel with each other or the RD direction. As shown in Figure 6, the UFG bundles at the upper-left corner and at the lower-right corner have the same <110> zone axis (Figure 6b,c) and a misorientation of 36.07°, which might be evolved from different initial CGs. The SAED patterns in Figure 6a are calibrated based on the magnetic declination of TEM. In order to coordinate this orientation relationship, some UFG grains are greatly bent, which are realized by the geometrically necessary dislocations at the GBs. Moreover, the GBs are not lying on the {111} planes. This may be caused by the cross slip of a large number of screw dislocations during the rolling process.

### 3.2. Microstructure of Annealed ARB-Processed Cu

**Figure 7:**
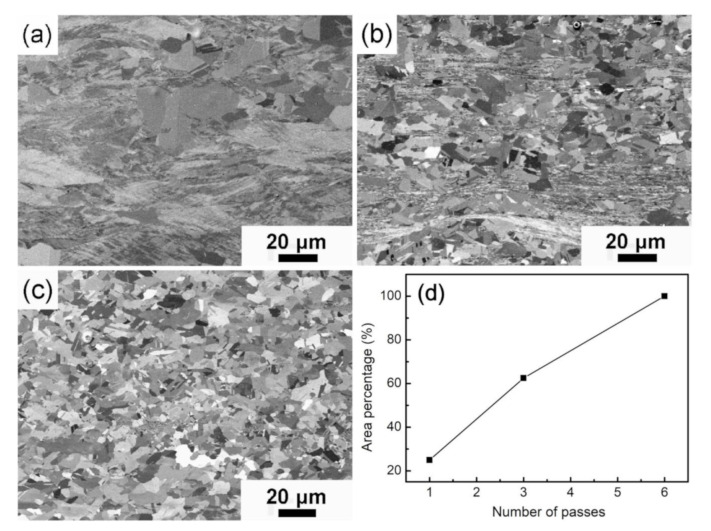
Microstructures of Cu ARBed for (**a**) 1 pass, (**b**) 3 passes, and (**c**) 6 passes after annealing for 60 min at 300 °C, and (**d**) the area percentage of recrystallized grains.

Figure 7 represents SEM images of ARB-processed Cu after annealing at 300 °C for 60 min. Evident changes were observed in the microstructure of plastically deformed specimens. After annealing, Cu ARB-processed for one pass exhibits a mixed microstructure of a small amount of recrystallized grains and a large number of deformed substructures (Figure 7a). Partial recrystallization with an area of ~25% (Figure 7d) occurred in high-strain regions. It should be noted that abnormal grain growth occurred in ARB-processed Cu after one pass. Figure 7b shows that the recrystallization area of ARB-processed Cu after three passes increased to ~62.5%, and the size of the recrystallized grains ranged from 5 to 15 μm. Recrystallization takes place at the interface with high strain energy prior to the other regions, suggesting a discontinuous recrystallization mechanism. For ARB-processed Cu after six passes, the newly recrystallized grains completely replaced the deformed microstructure. Moreover, the size of the recrystallized grains is approximately 5–10 μm, which is finer than that of the initial CG Cu (50 μm). Meanwhile, numerous annealing twins are formed in the recrystallized grains.

In addition to interface, discontinuous recrystallization can occur in regions with high dislocation density and cube texture. It is reported that cube-oriented grains have a higher growth rate than other orientations [36,37]. Thus, the formation of cube orientation results in discontinuous recrystallization and abnormal grain growth in Cu ARB-processed for one pass (Figure 7a). Compared with Cu prepared by equal-channel angular pressing [38], the recrystallized grains in ARB-processed Cu are not uniform due to the inhomogeneous deformed microstructure (Figure 2c). The heterogeneous deformed microstructure can be attributed to the inconsistent deformation in the matrix due to uneven distribution of shear forces along the thickness direction during ARB processing [39].

### 3.3. Mechanical Properties

**Figure 8:**
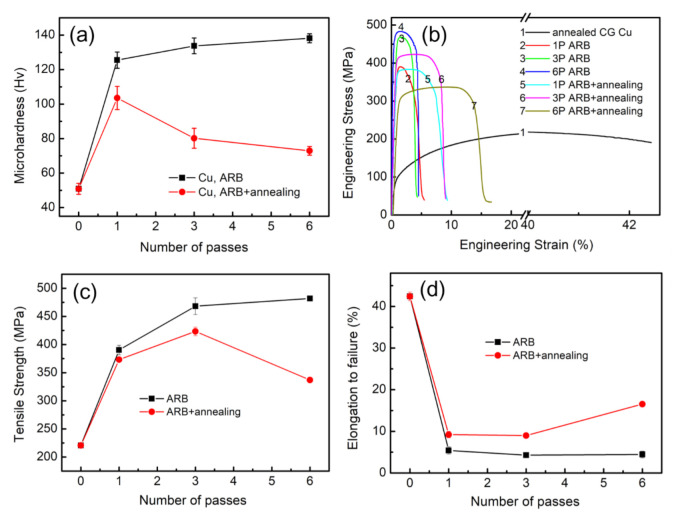
Microhardness (**a**), engineering stress–strain curves (**b**), tensile strength (**c**), and elongation to failure (**d**) of as-ARBed and as-annealed Cu.

Figure 8a presents the microhardness of ARB-processed Cu before and after annealing (at 300 °C for 60 min). The initial CG Cu is also included for comparison. After one pass, the microhardness of ARB-processed Cu was significantly increased due to grain refinement and strain hardening and reached a value of 115.17 Hv, which is higher than the annealed CG Cu (50.88 Hv). Then, the microhardness showed a slight increase with an increasing number of ARB passes. After six ARB passes, the microhardness reached a maximum value of 138.13 Hv, which is 2.7 times higher than that of as-received CG Cu. However, the microhardness decreased due to annealing at 300 °C for 60 min. In addition, the decrease in microhardness exhibited a direct relationship with the number of ARB passes. For instance, the microhardness of ARB-processed Cu with six passes decreased by half to 72.89 Hv.

Figure 8b presents the engineering stress–strain curves of ARB-processed Cu before and after annealing at 300 °C for 60 min. The indexes of mechanical strength and plasticity are listed in Table 3. The initial CG Cu showed the ductile behavior with an elongation to failure of 42.4%. However, the tensile strength of initial CG Cu is extremely low, as shown in Figure 8b (curve 1). A significant increase in strength, accompanied by a loss of ductility, was observed due to ARB deformation. Moreover, the necking phenomenon occurs immediately after yielding (curves 2, 3, and 4 in Figure 8b). Such necking instability is due to the lack of strain strengthening and dislocation storage after severe plastic deformation [27,38,40]. Furthermore, the tensile strength of ARB-processed Cu was reduced after annealing, whereas the failure elongation, corresponding to the ductility, was significantly increased, as shown in Figure 8b (curves 5, 6, and 7). These results reveal that strain hardening was remarkably recovered due to recovery and recrystallization of the deformed microstructure.

Figure 8c presents the relationship between the tensile strength of ARB-processed Cu and the number of ARB passes. After one ARB pass, the tensile strength of ARB-processed Cu increased from 220 to 390 MPa. Additionally, the tensile strength reached the maximum value of 482 MPa after six ARB passes, which is 2.2 times higher than that of CG Cu. However, the increase in strength and microhardness is accompanied by a significant decrease in ductility, as shown in Figure 8d. After one ARB pass, the elongation of ARB-processed Cu decreased from 42.43% to 5% due to the introduction of a large number of dislocations during the rolling process. It is worth mentioning that the mechanical behavior of ARB-processed Cu is consistent with previously reported ARB-processed metals [16] and other UFG materials [17,18,19,20], experiencing severe plastic deformation [41]. After annealing at 300 °C for 60 min, the tensile strength of ARB-processed Cu gradually decreased, whereas the ductility of the samples improved due to recovery and recrystallization. After one ARB pass, annealed ARB-processed Cu showed a recrystallization percentage of ~25%, but the deformed grains exhibited a relatively high strain energy state. Therefore, the strength slightly decreased, and the elongation recovered to 9.21% compared with that of ARB-processed Cu without annealing. After three ARB passes, annealed Cu demonstrated a tensile strength of 423 MPa and an elongation to failure of 8.98%. It is worth mentioning that the bimodal microstructure, with ultrafine grains and recrystallized grains, resulted in an optimal combination of strength and ductility. After six ARB passes, annealed Cu showed complete recrystallization and formation of new grains with a low density of dislocation. The tensile strength of ARB-processed Cu (six passes) decreased from 482 to 337 MPa after annealing, whereas the elongation to failure increased to 16.5%.

### 3.4. Fractography

**Figure 9:**
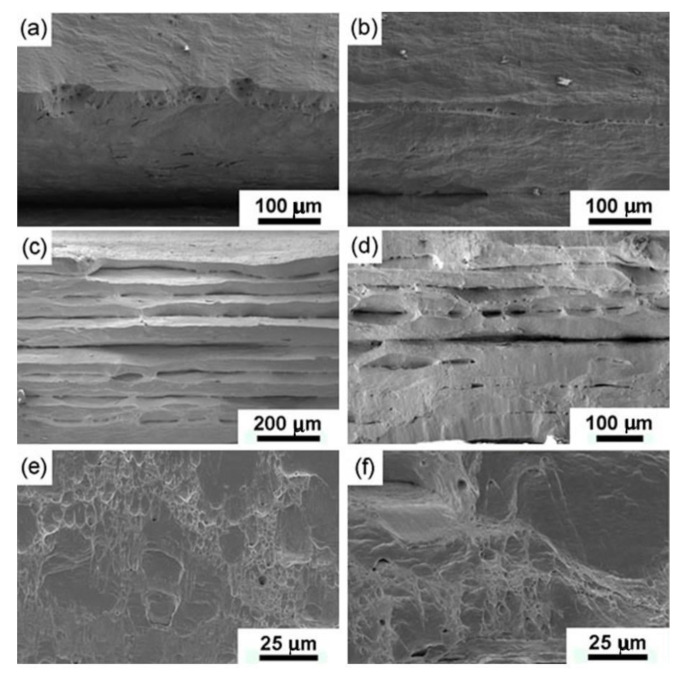
SEM micrographs of tensile fracture surfaces of Cu ARBed for (**a**) 1 pass, (**c**) 3 passes, and (**e**) 6 passes, and fracture surfaces of Cu ARBed for (**b**) 1 pass, (**d**) 3 passes, and (**f**) 6 passes after annealing 60 min at 300 °C.

Figure 9 shows the fractured surface of ARB-processed Cu before and after annealing at 300 °C for 60 min. The fractured surface of ARB-processed Cu exhibits elongated dimples, shear zones, and gross tearing, as shown in Figure 9a,c,e. As mentioned earlier, ARB-processed Cu showed a brittle fracture at the macroscopic level. The cracks originated from unbonded interfaces and matrix and rapidly spread under the action of applied tensile stress (Figure 9c). Necking is evidently formed within the individual Cu layers. Therefore, the SEM in Figure 9c revealed a delamination fracture due to weak bonding at the interface. Moreover, few shallow dimples in the matrix and interfacial region indicate a microvoid coalescence fracture. Thus, the as-ARBed specimens demonstrate ductile fracture at a microscopic level. The interface bonding improved after six ARB passes; however, there still existed some interfaces without metallurgical bonding. Quantitative evaluation of the interface without metallurgical bonding is hard because only high-resolution TEM can give the exact answer. It is hard for high-resolution TEM to obtain a statistic value. After annealing at 300 °C for 60 min, the interface between layers was healed, and the fracture occurred by a moderate amount of necking, which led to the ductile fracture of annealed ARB-processed Cu samples. The fracture morphology of this sample is dominated by dimples and shear zones (Figure 9f).

## 4. Discussion

For most engineering structural materials, it is ideal to simultaneously possess both high strength for carrying more load and high ductility for attaining higher toughness. Unfortunately, the strength and ductility of a material are generally trade-offs with each other, seldom coexisting. Like the ends of a teeterboard, elevating one has to lower the other. That is, strength and ductility consume each other: strengthening comes at the expense of plasticity and vice versa. This is the well-known so-called strength–ductility paradox, which exists universally in nature and is seldom broken [42,43,44,45,46,47].

**Figure 10:**
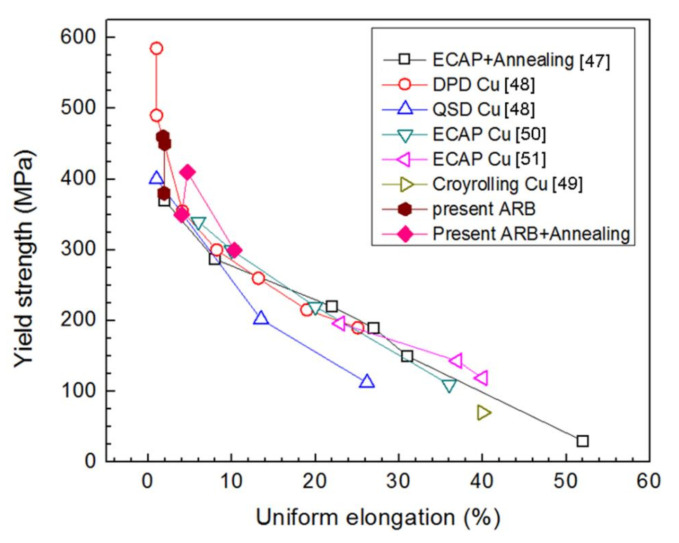
Yield strength versus uniform elongation of bimodal Cu prepared by dynamic plastic deformation (DPD), quasi-static deformation (QSD) [48], cryorolling [49], ECAP [47,50,51], ARB, and subsequent annealing.

Figure 10 illustrates the yield strength versus uniform elongation of bimodal Cu prepared by dynamic plastic deformation (DPD), quasi-static deformation (QSD) [48], cryorolling [49], ECAP [47,50,51], ARB, and subsequent annealing. Here, we compare the uniform elongation values, but not the elongation to failure, from different groups because our previous studies indicate that the former is not affected by tensile specimen size or geometry [52,53]. One can see our data points are located at the same region with data from the literature.

As summarized in Figure 10 for Cu, uniform elongation for CG Cu without cast artifacts is about 51%. Conventional strengthening mechanisms, including grain refinement and deformation, routinely enhance yield strength at the expense of ductility, following the strength–ductility paradox. NS and UFG Cu prepared by DPD and ECAP as well as cryorolling could have more than 5–10 times higher yield strength than CG Cu but disappointing low uniform elongation (<5%). Annealing enhances the uniform elongation with a concomitant decrease in yield strength. That is, σ_0.2_–ε_ue_ of the Cu samples exhibits approximately linear relationships: σ_0.2_ and ε_ue_ are inversely proportional to each other; that is, lowering strength increases ductility and vice versa. The strength–ductility paradox of metals and alloys arises from dislocation-slip dominated plastic deformation. Conventional strengthening mechanisms, including grain refinement and deformation, routinely increase the yield strength by increasing the critical shear stress for slip initiation, while ductility is related not only to dislocation nucleation but also more closely to slip kinetics, and is weakened more or less by the traditional strengthening mechanisms. Grain boundaries and deformation-induced dislocation cells cut the dislocation slip and multiplication and, therefore, reduce ductility. Therefore, dislocation-controlled plastic deformation determines the existence of the strength–plasticity dilemma.

Based on Hart’s theory [54], during tension, necking instability occurs when:

(1) Θ≤σ(1−m),

where Θ is the strain hardening rate, equal to ∂σ/∂ε; *m* is the strain rate sensitivity, equal to ∂lnσ/∂ln$\overset{\cdot }{\varepsilon }$; and σ, ε, and $\overset{\cdot }{\varepsilon }$ are true stress, true strain, and strain rate. Both high Θ and *m* are important for high tensile ductility because they can help delay the necking and prolong the elongation. Strain hardening (i.e., dynamic strengthening during tension) mainly results from interactions between dislocation and other lattice defects as well as itself, and *m* reflects a thermally activated mechanism of slip and relates to the flow stress activation volume V*. The *m* value of metals is usually much smaller than Θ when they deform quasi-statically at room temperature [55]. Therefore, strain hardening capability is import and determines the overall tensile ductility. Refining the CG down to NS and UFG regions takes away the space for dislocation accumulation and multiplication, and the high density of the boundaries as dislocation sinks makes the strain hardening null.

Great efforts have been made to enhance poor ductility, and more or less successes have been achieved until now [42,43,44,45,46,47]. However, the ductility of NS and UFG materials is still not over that of CG Cu (i.e., the strength–ductility paradox persists). It seems that the ductility of the CG counterpart is the extreme limit, which is difficult to exceed. The traditional solution in the literature is to make a compromise among the contradicting properties by combining their corresponding favorable structures together. For instance, both moderate strength and ductility are achieved in bimodal and gradient nanograined Cu because NS grains contribute to strength and CGs to ductility [27,55,56]. In fact, the compromise only makes a balance between the contradicting properties and does not solve the contradiction from the root. More recently, a back stress between UFG and CG regions based on a dislocation piling-up model was proposed to explain the origins of high strain hardening and ductility of bimodal and heterogeneous Ti and other metals [57,58,59,60].

In Figure 8b, our ARBed Cu has post-necking elongation smaller than 5%, which is much smaller than ECAPed Cu with post-necking elongation larger than 15% [61]. The reason for such evident difference is the weak bonding at the interface of ARBed Cu, which has been verified by SEM observation of the fracture surface in Figure 9. Our previous investigation indicates that reducing the thickness of tensile specimens of Cu can reduce the post-necking elongation significantly due to sample geometric effect on necking [52,53]. Moreover, in Figure 9c, the ARB-processed specimens show the formation of brittle fractures at the macroscopic level without annealing. The identification of manufacturing imperfections of such high-strength UFG metals and alloys could potentially be an interesting topic for both academic and industrial purposes. In this regard, many vibration-based techniques have been proposed recently. For example, Civera et al. reported a Gaussian process for manufacturing an imperfection identification of a pultruded glass fiber reinforced polymers (GFRP) thin-walled profile [62].

## 5. Conclusions

Ultrafine-grained copper sheets were fabricated using the ARB process at room temperature. Subsequently, the ARB-processed specimens were annealed at 300 °C for 60 min in order to optimize the mechanical properties of strength and ductility. The effects of the ARB process and the subsequent annealing on the microstructural evolution and mechanical properties of Cu were investigated. The conclusions are summarized as follows:

1. The lamellar UFGs are formed along the rolling direction, and the fraction of high-angle grain boundaries is improved by increasing the number of ARB passes. After six passes, the homogeneous microstructure throughout the thickness is obtained. The average boundary spacing along the normal direction is about 200–300 nm.
2. The more rolling passes there are, the greater the cumulative strain is, and the higher the microhardness and tensile strength become; however, the ductility becomes worse due to the absence of work hardening. The ARB-processed specimens fractured in a brittle manner.
3. Discontinuous recrystallization occurs in ultrafine-grained copper during annealing. That is, recrystallization at the interface with high strain energy is prior to that in the matrix. Increasing the cumulative strain enhances the rate of recrystallization. For Cu ARB-processed for six passes after annealing, recrystallization is complete. The grain size of recrystallized grain is approximately 5–10 μm.
4. After annealing, the microstructure and properties have relative restoration as a result of recovery and recrystallization. The interfaces between layer and layer have obvious healing during annealing. Annealing treatment reduces the microhardness and tensile strength but improves the ductility and strain hardening of UFG Cu. The fracture mode of as-annealed specimens is ductile fracture. The fracture morphologies are dominated by dimples and shear zones. Because ARB is a ready-made deformation technique in the industry, the preparation of our UFG Cu has a simple operation, low cost, and easy commercialization.

## Figures and Tables

**Table 1:** Measured chemical composition (wt. %) of as-received pure Cu by inductively coupled plasma emission spectrometer and ONH analyzers.

Element	wt. %	Element	wt. %
Pb	0.001	Sn	0.002
Fe	0.004	Ni	0.002
Bi	0.001	Zn	0.003
Sb	0.002	P	0.002
As	0.002	S	0.004

**Table 2:** List of {100}, {110}, and {111} polar figures of ARB-processed Cu for 1, 3, and 6 passes.

Samples	{100} Pole	{110} Pole	{111} Pole	Ruler
ARB for 1 pass	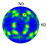	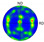	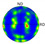	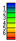
ARB for 3 passes	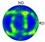	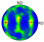	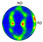	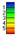
ARB for 6 passes	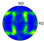	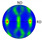	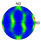	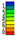

**Table 3:** List of yield strength (YS), ultimate tensile strength (UTS), uniform elongation ε_ue_, and elongation to failure ε_ef_ of as-ARBed and as-annealed Cu samples.

Samples	YS, MPa	UTS, MPa	ε_ue_, %	ε_ef_, %
ARB 1 pass	380	390	1.9	5.4
ARB 3 passes	450	468	2.0	4.3
ARB 6 passes	460	482	1.8	4.5
Annealed ARB 1 pass	350	363	4.0	9.2
Annealed ARB 3 pass	410	423	4.7	9.0
Annealed ARB 6 pass	300	337	10.3	16.5
CG Cu	70	220	40	42.4
